# Supplementary material for: The Effects of Artificial Diets Containing Free Amino Acids Versus Intact Proteins on Biomarkers of Nutrition and Deformed Wing Virus Levels in the Honey Bee
Source: Insects. 2025 Apr 2;16(4):375. doi: 10.3390/insects16040375 (PMC12027968; doi:10.3390/insects16040375)
Supplement: Supplementary file 1 [file insects-16-00375-s001.zip › insects-3507688-supplementary.pdf]

Table S1. Proximal bromatological analysis of the composition, TAA, Apitir® and Ultrabee® diets. The analysis of intact protein and amino acid content was conducted using the Kjeldahl method to measure nitrogen, followed by a conversion factor.

Main Components	***TAA***	***Apitir ®***	***Ultrabee ®***
Dry matter (g/100g)	0	95.6	95.8
Crude protein/amino acids (g/100g)	3.5	21.5	59.5
Crude fat (g/100g)	0.7	4.5	< 5
Ash (g/100g)	0.14	3	< 6
Amino acid profiles			
Leucine (g/100g)	0.59	1.5	7.8
Isoleucine (g/100g)	0.525	0.98	2.25
Arginine (g/100g)	0.523	1.08	2.28
Valine (g/100g)	0.469	1.16	2.66
Glutamic acid (g/100g)	0.441	3.28	11.4
Lysine (g/100g)	0.439	1.64	5.44
Phenylalanine (g/100g)	0.413	0.93	3.29
Aspartic acid (g/100g)	0.399	2.18	4.45
Cysteine + Cystine (g/100g)	0.363	0.33	0.89
Threonine (g/100g)	0.357	1.05	2.09
Hydroxyproline (g/100g)	0.345	<0.2	<0.2
Proline (g/100g)	0.345	0.81	4.39
Serine (g/100g)	0.315	1.05	3
Alanine (g/100g)	0.267	1.2	4.28
Histidine (g/100g)	0.233	0.46	1.22
Glycine (g/100g)	0.225	0.99	1.82
Methionine (g/100g)	0.224	0.49	1.14
Tryptophan (g/100g)	0.204	0.26	0.48
Tyrosine (g/100g)	0.036	0.72	2.49
Ornithine (g/100g)		<0.05	<0.05

Table S2. Primers used for qPCRs**.** Primers' names include the forward (F) and reverse (R) orientations and the base-pair length. All primer pairs had an efficiency of approximately 100% in our experimental system.

Target	Gene ID	Sequence (5' to 3')	Primer size	Primer Tm	Amplicon size (bp)	Reference
vg	GB49544	AGTTCCGACCGACGACG	vg-F17	60.6	63	[68]
		TTCCCTCCCACGGAGTCC	vg-R18	61.1		
mrjp1	GB55205	TGACCAATGGCATGATAAGATTTT	mrjp-F24	57.5	98	[53]
		GACCACCATCACCGACCT	mrjp-R18	59.9		
rps5	GB45730	AATTATTTGGTCGCTGGAATTG	rps5-F22	56.7	115	[67]
		TAACGTCCAGCAGAATGTGGTA	rps5-R22	57.4		
**DWV**	MG831204	GAGATTGAAGCGCATGAACA	DWV-F20	55.9	130	[67]
		TGAATTCAGTGTCGCCCATA	DWV-R20	56.7		

Table S3. Probability values from paired comparisons of nutritional and age groups on the relative values of *vg*, *mrjp1*, and DWV**.** All the targets were analyzed using the Mann-Whitney test. Significant p- values (are indicated in bold case.

		Vg		***Mrjp1***		**DWV**	
Var1	vs Var 2	U	p	U	p	U	p
W0-NEBs	W1-Sugar	58	**0.006**	20.	0.355	39.	0.895
W0-NEBs	W1-TAA	39	0.895	32.	0.7	72.	**0.001**
W0-NEBs	W1-Apitir	28	0.441	33.	0.916	67.	**0.003**
W0-NEBs	W1-UBee	44.	0.186	25.	0.728	21.	0.48
W0-NEBs	W2-Sugar	4.	**0.004**	0.	**0.002**	57.	**0.043**
W0-NEBs	W2-Apitir	19.	0.309	15.	0.54	27.	**0.013**
W0-NEBs	W2-UBee	29.	0.386	14.	0.38	5.	**0.045**
W0-NEBs	W3-Sugar	3.	**0.021**	0	**0.003**	45.	**0.003**
W0-NEBs	W3-UBee	2.	0.099	0	**0.014**	18.	**0.034**
W1-Sugar	W1-TAA	0.	**0.001**	8.	**0.007**	72.	**0.0005**
W1-Sugar	W1-Apitir	7.	**0.009**	19.	0.172	67.	**0.003**
W1-Sugar	W1-UBee	56.	**0.001**	38.	0.247	22.	0.556
W1-Sugar	W2-Sugar	32.	0.643	0.	**0.002**	56.	0.054
W1-Sugar	W2-Apitir	24.	**0.014**	24.	**0.014**	27.	**0.013**
W1-Sugar	W2-UBee	40.	**0.003**	21.	0.884	7.	0.09
W1-Sugar	W3-Sugar	19.	0.610	0.	**0.003**	45.	**0.003**
W1-Sugar	W3-UBee	12.	0.296	1.	**0.025**	18.	**0.034**
W1-TAA	W1-Apitir	13.	**0.027**	18.	0.083	58.	**0.006**
W1-TAA	W1-UBee	41.	0.315	11.	**0.03**	0.	**0.002**
W1-TAA	W2-Sugar	0.	**0.001**	1.	**0.001**	64.	**0.001**
W1-TAA	W2-Apitir	15.	0.782	18.	0.405	0.	**0.014**
W1-TAA	W2-UBee	27.	0.549	6.	**0.028**	0.	**0.007**
W1-TAA	W3-Sugar	0.	**0.014**	0.	**0.003**	0.	**0.003**
W1-TAA	W3-UBee	2.	**0.014**	0.	**0.013**	5.	0.433
W1-Apitir	W1-UBee	53.	**0.004**	25.	0.728	1.	**0.003**
W1-Apitir	W2-Sugar	7.	**0.015**	0.	**0.003**	48.	0.093
W1-Apitir	W2-Apitir	22.	0.041	22.	**0.041**	10.	0.683
W1-Apitir	W2-UBee	31.	0.107	13.	0.306	28.	**0.007**
W1-Apitir	W3-Sugar	4.	**0.042**	0.	**0.003**	18.	0.77
W1-Apitir	W3-UBee	2.	0.117	0.	**0.014**	12.	0.296
W1-UBee	W2-Sugar	56.	**0.001**	0.	**0.003**	5.	**0.014**
W1-UBee	W2-Apitir	10.	0.909	21.	**0.017**	18.	**0.02**
W1-UBee	W2-UBee	14.	0.570	13.	0.465	3.	0.055
W1-UBee	W3-Sugar	0.	**0.008**	0.	**0.004**	30.	**0.006**
W1-UBee	W3-UBee	0.	**0.040**	0.	**0.017**	12.	**0.046**
W2-Sugar	W2-Apitir	24.	**0.014**	18.	**0.02**	18.	0.221
W2-Sugar	W2-UBee	40.	**0.003**	30.	**0.006**	0.	**0.007**
W2-Sugar	W3-Sugar	10.	0.450	24.	0.1	29.	0.188
W2-Sugar	W3-UBee	9.	0.794	18.	**0.02**	16.	**0.037**
W2-Apitir	W2-UBee	9.	0.655	15.	**0.025**	12.	**0.034**
W2-Apitir	W3-Sugar	12.	**0.034**	15.	**0.025**	9.	0.655
W2-Apitir	W3-UBee	9.	0.083	9.	**0.05**	6.	0.083
W2-UBee	W3-Sugar	0.	**0.014**	25.	**0.009**	0.	**0.014**
W2-UBee	W3-UBee	0.	0.053	0.	**0.025**	8.	0.064
W3-Sugar	W3-UBee	6.	0.355	14.	0.053	10.	0.053
